# Community composition of root-associated fungi in a *Quercus*-dominated temperate forest: “codominance” of mycorrhizal and root-endophytic fungi

**DOI:** 10.1002/ece3.546

**Published:** 2013-04-05

**Authors:** Hirokazu Toju, Satoshi Yamamoto, Hirotoshi Sato, Akifumi S Tanabe, Gregory S Gilbert, Kohmei Kadowaki

**Affiliations:** 1 Graduate School of Global Environmental Studies, Kyoto UniversitySakyo, Kyoto, 606-8501, Japan; 2 Graduate School of Human and Environmental Studies, Kyoto UniversitySakyo, Kyoto, 606-8501, Japan; 3 National Research Institute of Fisheries Science, Fisheries Research AgencyFukuura, Kanazawa, Yokohama, Kanagawa, 236-8648, Japan; 4 Environmental Studies Department, University of CaliforniaSanta Cruz, California, 95064

**Keywords:** 454 next-generation sequencing, dark septate endophytes, fungal communities, metagenomics, mycorrhizae, network theory

## Abstract

In terrestrial ecosystems, plant roots are colonized by various clades of mycorrhizal and endophytic fungi. Focused on the root systems of an oak-dominated temperate forest in Japan, we used 454 pyrosequencing to explore how phylogenetically diverse fungi constitute an ecological community of multiple ecotypes. In total, 345 operational taxonomic units (OTUs) of fungi were found from 159 terminal-root samples from 12 plant species occurring in the forest. Due to the dominance of an oak species (*Quercus serrata*), diverse ectomycorrhizal clades such as *Russula*, *Lactarius*, *Cortinarius*, *Tomentella*, *Amanita*, *Boletus*, and *Cenococcum* were observed. Unexpectedly, the root-associated fungal community was dominated by root-endophytic ascomycetes in Helotiales, Chaetothyriales, and Rhytismatales. Overall, 55.3% of root samples were colonized by both the commonly observed ascomycetes and ectomycorrhizal fungi; 75.0% of the root samples of the dominant *Q. serrata* were so cocolonized. Overall, this study revealed that root-associated fungal communities of oak-dominated temperate forests were dominated not only by ectomycorrhizal fungi but also by diverse root endophytes and that potential ecological interactions between the two ecotypes may be important to understand the complex assembly processes of belowground fungal communities.

## Introduction

In terrestrial ecosystems, diverse mycorrhizal fungi are associated with plant roots, transporting soil nutrients to their plant hosts (Allen 1991; Smith and Read 2008). In general, mycorrhizal fungi enhance the growth and survival of their host plants which in return provide carbohydrates to the fungi (Högberg et al. 2001; Högberg and Högberg 2002). However, the performance benefits and energetic costs of mycorrhizal symbiosis for a plant vary among symbiotic fungal species or strains (Gao et al. 2001; Nara 2006; Hoeksema 2010; Johnson et al. 2012), and both plants and fungi show strain- or species-specific compatibility with their symbionts (Bruns et al. 2002; Sato et al. 2007; Tedersoo et al. 2008; Davison et al. 2011). Such variation in specificity and impacts in plant–fungal symbioses will affect how phylogenetically diverse fungi can coexist in a community, and hence we need to understand the community composition of fungi associated with roots as well as their preference for host plants at a community-wide scale.

In the Northern Hemisphere, temperate forests are generally dominated by trees in the Fagaceae and Pinaceae. Species in these plant families form mycorrhizae with various phylogenetic clades of ectomycorrhizal fungi (Jumpponen et al. 2010; Bahram et al. 2012; Sato et al. 2012a,b; Tedersoo et al. 2012). These ectomycorrhizal fungi extend extraradical mycelia into soil and transport soil nitrogen and phosphorus to their host plants (Finlay and Read 1986; Cairney 2005; Wu et al. 2012). In addition, some ectomycorrhizal fungi protect host roots from pathogenic fungi or nematodes (Azcón-Aguilar and Barea 1997; Borowicz 2001). Through such impacts, ectomycorrhizal fungi play essential roles in the growth and survival of oaks and pines, presumably affecting the competitive ability of their hosts in local communities.

Roots of oak and pine trees can be colonized by symbionts in addition to ectomycorrhizal fungi, including arbuscular mycorrhizal fungi (Dickie et al. 2001) and various clades of root-endophytic fungi (Girlanda et al. 2002; Wagg et al. 2008; Kernaghan and Patriquin 2011; Reininger and Sieber 2012). Recent focus on the “hidden diversity” of root-endophytic fungi has uncovered their prevalence in various types of terrestrial ecosystems and their probable benefit to host plants (Jumpponen and Trappe 1998; Jumpponen 2001; Newsham 2011; Porras-Alfaro and Bayman 2011). For example, various clades of “dark septate endophytes” can transform organic nitrogen to inorganic forms in the rhizosphere, making the nutrient available to their hosts (Upson et al. 2009; Newsham 2011). Importantly, while ectomycorrhizal fungi generally associate with a narrow range of host taxa (Sato et al. 2007; Tedersoo et al. 2008), many of root-endophytic fungi have broad host ranges (Walker et al. 2011; Knapp et al. 2012; Mandyam et al. 2012). Therefore, ectomycorrhizal and root-endophytic fungi may contribute differentially to the dynamics of forest communities. A comparative assessment of the community structures of ectomycorrhizal and root-endophytic fungi in the same ecosystem is needed to help understand how each contributes to shaping forest tree communities through plant–fungal interactions.

In this study, we describe the community composition of root-associated fungi in an oak-dominated temperate forest in Japan based on 454 pyrosequencing of ribosomal internal transcribed spacer (ITS) sequences. We describe the community structure of root-associated fungi in terms of (1) taxonomy, (2) habitat preference (plant root vs. soil), and (3) host-plant preference. First, molecular identification from sequence matching and a supplemental phylogenetic analysis was used to determine whether each of the commonly observed fungi were from clades of fungi known to be mycorrhizal or known to include root endophytes. Second, to infer the role of these root-associated fungi in providing plants with access to soil nutrients, we evaluated the prevalence of those fungi in rhizosphere soil. We predicted that ectomycorrhizal fungi would be common in soil because they form extraradical mycelia that extend away from the root (Finlay and Read 1986), whereas endophytic fungi would be found almost exclusively in root samples (Rodriguez et al. 2009). Third, we evaluated preference of the dominant fungal taxa for host plant species, expecting ectomycorrhizal fungi to show relatively high host preference (Sato et al. 2007; Tedersoo et al. 2008) and root-endophytic fungi a broader host range (Walker et al. 2011; Knapp et al. 2012; Mandyam et al. 2012). Finally, we examined the degree to which ectomycorrhizal and root-endophytic fungi co-occur within roots.

## Materials and Methods

### Sampling

Root samples were collected in a temperate secondary-growth forest on Mt. Yoshida, Kyoto, Japan (35°02′N, 135°47′E; parent material = chert) on 17–18 August 2011. In the study site, a deciduous oak tree, *Quercus serrata*, is dominant, while broad-leaved evergreen trees such as *Ilex pedunculosa* (Aquifoliaceae) and *Q. glauca* co-occur at the canopy layer. In a 13 m-by-13 m plot, 196 sampling positions were set at 1-meter intervals. At each sampling position, two 2-cm segments of terminal root were collected from the upper part of the A horizon (3 cm below the soil surface). Terminal roots colonized by ectomycorrhizal associates of *Quercus* species have a characteristic branching morphology, whereas unbranched root samples are typical of roots colonized by other types of mycorrhizae only by endophytes or pathogens. We collected terminal-root samples indiscriminately in terms of root morphology or apparent mycorrhizal type so that the samples as a whole should represent the relative frequency of plant–fungal associations in the horizon at the study plot (Nielsen and Bascompte 2007; Montesinos-Navarro et al. 2012).

To examine how much the root-associated fungal community extends away from roots into rhizosphere soil, we sampled 1 cm^3^ soil surrounding root samples, collected at 2-m intervals across the 169-m^2^ study site (49 samples; Fig. S1). Both root and soil samples were immediately preserved in absolute ethanol upon collection and stored at −20°C in the laboratory.

### DNA extraction, PCR, and pyrosequencing

One terminal root was randomly chosen from each of 196 sampling positions and subjected to the DNA extraction, PCR, and sequencing. All soil was carefully removed from the root samples by placing the roots in 70% ethanol with 1-mm zirconium balls, and then shaking the sample tubes 15 times per second for 2 min using TissueLyser II (Qiagen, Venlo, Netherlands) (Fig. S2). Samples were frozen at −20°C and then pulverized by shaking on the TissueLyser II with 4-mm zirconium balls 20 times per second for 3 min. We extracted plant and fungal DNA from each root sample using a cetyl trimethyl ammonium bromide (CTAB) method as detailed elsewhere (Sato and Murakami 2008). To extract DNA from soil samples, we carefully removed root and plant debris, and then extracted DNA from 150 mg dried soil per sample, using the CTAB method.

As the concentration of PCR products to be pooled for massively parallel pyrosequencing must be equalized among tag-encoded samples, a two-step (nested) PCR was used to saturate the concentration of the PCR amplicons of each sample. For each root sample, plant chloroplast *rbcL* sequences were amplified using the primers rbcL_rvF (5′-CCA MAA ACR GAR ACT AAA GC-3′) and rbcL_R1 (5′-CGR TCY CTC CAR CGC AT-3′) with the buffer system of Ampdirect Plus (Shimadzu, Kyoto, Japan) and BIOTAQ HS DNA Polymerase (Bioline, London, U.K.). PCR was conducted under a temperature profile of 95°C for 10 min, followed by 30 cycles of 94°C for 20 sec, 50°C for 30 sec, and 72°C for 40 sec, and final extension at 72°C for 7 min. The PCR product of each root sample was subjected to the second PCR amplification of 0.5-kb *rbcL* gene fragment using the rbcL_rvF primer fused with the 454 pyrosequencing Adaptor A (5′-CCA TCT CAT CCC TGC GTG TCT CCG ACT CAG-3′) and the 8-mer molecular ID (Hamady et al. 2008) of each sample, and the reverse primer rbcL_R2 (5′-CCY AAT TTT GGT TTR ATR GTA C-3′) fused with the 454 Adaptor B (5′-CCT ATC CCC TGT GTG CCT TGG CAG TCT CAG-3′). The second PCR was conducted with the buffer system of Taq DNA Polymerase with Standard Taq Buffer (New England BioLabs, Ipswich, MA) under a temperature profile of 95°C for 1 min, followed by 40 cycles of 94°C for 20 sec, 50°C for 30 sec, and 72°C for 40 sec, and final extension at 72°C for 7 min.

For each root and soil sample, the entire range of fungal ITS sequences were amplified using the fungus-specific high-coverage primer ITS1F_KYO2 (Toju et al. 2012) and the universal primer ITS4 (White et al. 1990). The PCR product of each root or soil sample was subjected to the second PCR step targeting ITS2 region using the universal primer ITS3_KYO2 (Toju et al. 2012) fused with the 454 adaptor A and each sample-specific molecular ID, and the reverse universal primer ITS4 fused with the 454 adaptor B. The first and second PCR steps of ITS region were conducted under the same buffer systems and temperature profiles as those of *rbcL*. All the *rbcL* and ITS amplicons of the second PCR steps were pooled and subjected to a purification process by ExoSAP-IT (GE Healthcare, Little Chalfont, U.K.) and QIAquick PCR Purification Kit (Qiagen). As instructed by the manufacturer, 454 pyrosequencing was performed on a GS Junior sequencer (Roche, Basel, Switzerland).

### Assembly of sequencing reads

Hereafter, we describe the pyrosequencing procedure as suggested by Nilsson et al. (2011). For the pyrosequencing reads output by GS Junior (DDBJ DRA: DRA000728), trimming of low-quality 3′ tails was conducted with a minimum quality value of 20. Of the 99,101 output reads, 76,818 reads (5112 *rbcL* and 71,706 ITS reads) passed the filtering process in which *rbcL* reads shorter than 400 bp and ITS reads with fewer than 150 bp excluding forward primer and molecular ID positions were discarded. *RbcL* and ITS reads were recognized by the primer position sequences and analyzed separately. For each gene, pyrosequencing reads were sorted by samples using the sample-specific molecular IDs. Molecular ID and forward primer sequences were removed before assembly. Denoising of the pyrosequencing data was performed based on the assembling of reads (see below; cf. Li et al. 2012), which did not depend on computationally intensive methods using flowgram data.

We assembled the sequence data using Assams v0.1.2012.03.14 (Tanabe 2012a), which is a highly parallelized extension of Minimus assembly pipeline (Sommer et al. 2007). For host plant *rbcL* gene, reads in each sample were assembled with a minimum cutoff similarity of 97% to remove pyrosequencing errors and then obtain the consensus *rbcL* gene sequence of each root sample. After the elimination of possible chimeras using the program UCHIME v4.2.40 (Edgar et al. 2011), the consensus sequences for root samples (within-sample consensus sequences) were further assembled across samples with a minimum similarity setting of 99.8%. These consensus sequences (among-sample consensus sequences) were compared to the reference *rbcL* sequences of the plants occurring at the study sites (AB729077–AB729106) to identify the host plant species of each root sample.

To process sequence data from the fungal ITS2 region of root and soil samples, reads were subjected to in silico detection and removal of chimeras (Edgar et al. 2011). In each sample, reads were assembled by Assams with a minimum similarity setting of 97% and then chimera reads were eliminated using the program UCHIME v4.2.40 (Edgar et al. 2011) with a minimum score to report chimera of 0.1. Of the 71,706 ITS reads, 1211 reads were discarded as chimeras, leaving 70,495 reads.

The within-sample consensus sequences represented by the 70,495 ITS reads were assembled at a cutoff similarity of 97%, and the resulting among-sample consensus sequences assigned as fungal operational taxonomic units (OTUs; Data S1). Of the 70,495 reads, 556 reads were singletons and were excluded from further analysis. Since OTU sequences reconstructed from a small number of sequencing reads could be susceptible to sequencing errors, only OTUs representing at least five reads in at least one sample were included in analyses (Data S1). Samples with fewer than 100 high-quality reads were eliminated, leaving 159 root and 38 soil samples. On average, 357.1 (SD = 80.3; *N* = 159) or 307.9 (SD = 123.6; *N* = 38) reads were obtained for each root or soil sample (Data S2).

### Molecular identification of fungi

As our samples potentially included not only ectomycorrhizal fungi but also diverse and poorly known root-endophytic and soil fungi, BLAST top-hit sequences in the NCBI database did not provide enough taxonomically informative matches, even when we eliminated NCBI-database sequences registered as “uncultured” fungi (Data S3). Similarly, comparison of our sequences to the UNITE database (Abarenkov et al. 2010; http://unite.ut.ee/), which includes high-quality ITS sequences of fruiting body specimens identified by experts and deposited in public herbaria, allowed identification of ectomycorrhizal OTUs to genus or species (Data S3). Unfortunately, many other OTUs did not match any UNITE database sequences (see low query coverage of the UNITE search in Data S3), making it difficult to identify fungi that were not ectomycorrhizal.

Therefore, to systematically infer the taxonomy of the OTUs, we used Claident v0.1.2012.03.14 (Tanabe 2012b), which integrates BLAST+ (Camacho et al. 2009) and NCBI taxonomy-based sequence identification engines as well as utilities to create BLAST databases of sequences with sufficient taxonomic information. Two BLAST databases were created using Claident and BLAST+, subsets of the “nt” database downloaded from NCBI ftp server (http://www.ncbi.nlm.nih.gov/Ftp/) on 8 February 2012. The first subset database (“genus” database) consisted of sequences identified at genus or species level (i.e., sequences not identifiable to the genus level were eliminated). The “class” database, then, contained sequences identified at class or lower taxonomic level (i.e., sequences unable to be identified to at least the class level were removed). Because only a small proportion of fungal sequences in public databases have been deposited with genus names (Abarenkov et al. 2010; Hibbett et al. 2011), the “genus” database is insufficient for the identification of many of fungal OTUs in root or soil samples. Thus, the “class” database was used as well to complement the identification based on the “genus” database (see below).

In each “genus” or “class” database, sequences homologous to each query (OTU) sequence were searched with the aid of the “clidentseq” command of Claident. Identification of OTUs was subsequently performed by the “classigntax” command of Claident based on the lowest common ancestor (LCA) algorithm (Huson et al. 2007). The algorithm assigns each query to the lowest taxonomic level common to the homologous sequences (Huson et al. 2007). However, this algorithm is sometimes too conservative and a high proportion of fungal OTUs remain unidentified, because even rare sequences with erroneous taxonomic information in the NCBI database can interrupt identification. Therefore, each OTU was also identified using a “relaxed LCA algorithm”. In the relaxed LCA algorithm, inclusion of 10% of homologous sequences whose taxonomic information was inconsistent with that of the remaining 90% homologous sequences was tolerated. Thus, each sequence was given a taxonomic identification in three ways: the default LCA algorithm with the “genus” database (LCA/genus), the relaxed LCA algorithm with the “genus” database (relaxed-LCA/genus), and the default LCA algorithm with the “class” database (LCA/class). The final identification results (Data S4) were obtained by merging LCA/genus, LCA/class, and relaxed-LCA/genus results with priorities in this order, using the “clmergeassign” command in Claident.

### Molecular phylogeny of commonly observed fungi

For the 10 most common OTUs observed from roots but whose taxonomy was unidentified at genus level (Data S3), we conducted a molecular phylogenetic analysis to further infer the taxonomic identity of the OTUs. Multiple alignments of ITS sequences were performed using the program MAFFT v6.813b (Katoh et al. 2005), followed by elimination of ambiguously aligned nucleotide sites using GBlocks Server v0.91b (Castresana 2000). Best-fit substitution models for the aligned sequences were selected using the program Kakusan v4 (Tanabe 2011). Maximum likelihood phylogenies were inferred using the software Treefinder (Jobb et al. 2004) with the tool package Phylogears v1.5 (Tanabe 2008), whereby parallelized tree search bootstrapping was conducted.

### Data matrix for the analyses of habitat/host preference

We created a presence/absence community matrix of fungal OTUs for all 159 root and 38 soil samples. The number of sequencing reads varied among samples (106–635 reads), which could artificially generate variance in estimates of α-diversity among samples. To reduce this variance in the following habitat/host preference analyses, we excluded rare OTUs represented by less than 5% of the sample total reads. This resulting matrix (Data S5) then was based on 79 to 592 reads per sample after removing any OTUs identified as representing host plants or Metazoa (Data S4).

### Habitat preference

We evaluated whether each fungal OTU occurred preferentially in roots or soil. Habitat preference was evaluated using the multinomial species classification method (CLAM; Chazdon et al. 2011) implemented in the “clamtest” command of the “vegan” v2.0-2 package (Oksanen et al. 2012) of R (http://cran.r-project.org/). Each OTU was classified as showing statistically significant habitat preference to root samples, to soil samples, or as being commonly observed in both habitats, based on the “supermajority” rule (Chazdon et al. 2011).

### Host preference

To evaluate the host preference of fungal OTUs, we compiled a plant × fungal OTU matrix (Data S6) that shows the number of root samples in which each plant–fungal association was observed. Root species presence was based on the *rbcL* sequences and fungal OTU presence from the fungal presence/absence matrix (Data S5). Since the root samples were washed prior to the PCR and pyrosequencing, fungi detected from each root sample were considered physically connected to the plant tissue (“symbiosis” in the broad sense).

We tested host preference of respective OTUs by calculating the *d'* index of specialization of interspecific interactions (Blüthgen et al. 2007) using the “dfun” command of the “bipartite” v1.17 package (Dormann et al. 2009) of R. The *d'* index measures how strongly a plant species (a fungus) deviates from a random choice of interacting fungal partners (host plant partners) available. It ranges from 0 (extreme generalization) to 1 (extreme specialization) (Blüthgen et al. 2007). The observed *d'* measures were compared with those of randomized plant × fungus matrices, in which combinations of plants and fungal OTUs were randomized under “vaznull” model (Vázquez et al. 2007) using the bipartite package (10,000 permutations). For simplicity, we show the results of the 10 most common OTUs.

To visualize the overall architecture of the plant–fungal associations represented by the plant × fungus matrix (Data S6), the “gplot” command of the “sna” v2.2-0 package (Butts 2010) of R was used. The graph of plant–fungal associations illustrated how host-specific fungal OTUs and OTUs with broad host range were distributed within a web of symbiosis. Note that this does not represent the structure of “common mycelial network” (Nara 2006; Beiler et al. 2009), which should be analyzed based on plant–fungal interactions at individual level.

### Co-occurrence of fungal OTUs within roots

Patterns of the co-occurrence of multiple fungal OTUs within terminal roots were investigated. We calculated the proportion of root samples that were infected by multiple ecotypes of fungi (i.e., ectomycorrhizal, arbuscular mycorrhizal, or root-endophytic fungi; Data S4).

## Results

### Molecular identification and fungal diversity within each sample

**Figure 1:**
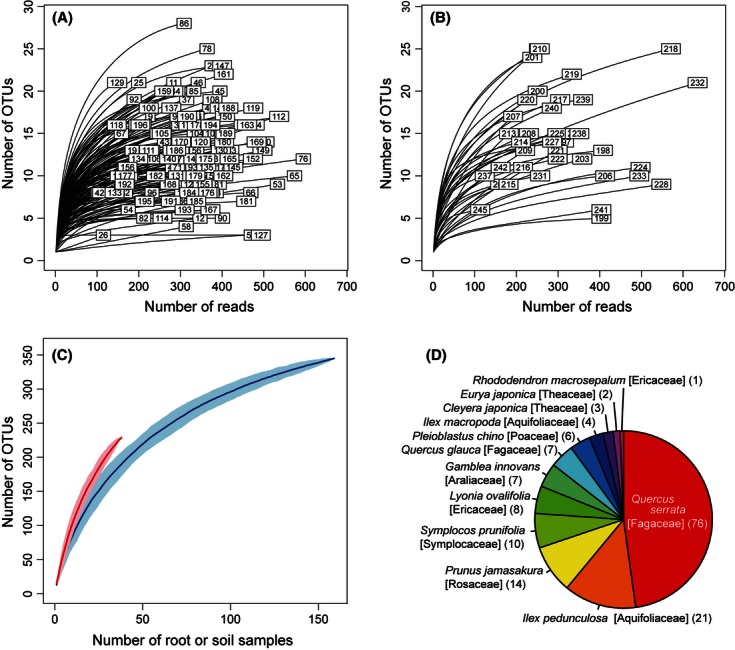
Diversity of fungi and host plants in the samples. (A) Rarefaction curve of OTUs in each root sample against the number of pyrosequencing reads excluding singletons. The numbers in boxes represents sample ID (Data S2). (B) Rarefaction curve of OTUs in each soil sample against the number of pyrosequencing reads excluding singletons. (C) Rarefaction curve of OTUs against root (blue) or soil (red) sample size. The shaded area represents the standard deviation (standard error of the estimate) obtained from 100 shuffling of sample-ID order. (D) Composition of host plant species identified by chloroplast *rbcL* sequences. The number of root samples was indicated in parentheses.

In total, we found 392 fungal OTUs from the root and soil samples (Data S2). Among those, 163 and 47 OTUs were found exclusively from root or soil samples, respectively, and 182 were common to both sample types. Among the 392 OTUs, 181 were ascomycetes, 108 basidiomycetes, two chytridiomycetes, and five glomeromycetes, while 96 fungal OTUs could not be identified to the phylum level. The mean number of OTUs observed in each sample did not significantly differ between root samples (12.9 OTUs [SD = 4.7]) and soil samples (14.5 OTUs [SD = 5.4]; Fig. 1A and B) after controlling for the number of sequencing reads per sample (generalized-linear model with quasi-Poisson error; *t*_1, 194_ = 1.9, *P* = 0.057). The mean number of arbuscular and ectomycorrhizal fungal OTUs in a sample was 1.9 (SD = 1.3, *N* = 159) for roots and 2.7 (SD = 1.8, *N* = 38) for soil. For both sample types, the total number of observed OTUs increased continuously with increasing sample size (Fig. 1C), reflecting the high diversity of belowground fungi.

### Community composition of root-associated fungi

The analysis of chloroplast *rbcL* gene sequences revealed that the 159 terminal-root samples represented 12 plant species (Fig. 1D). Among the plant species, *Q. serrata* was the most dominant, as expected by the dominance of the plant aboveground in the study site.

**Figure 2:**
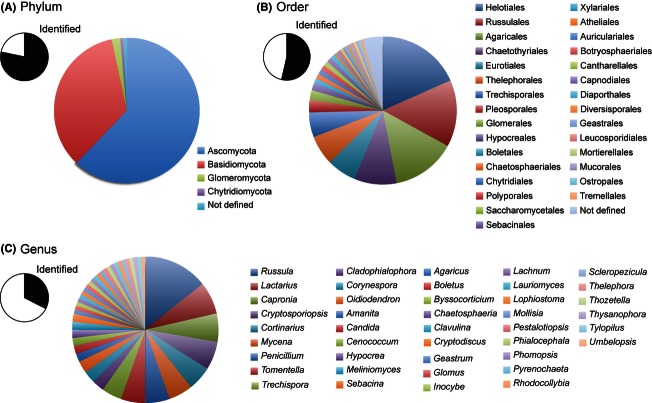
Community composition of root-associated fungi. (A) Phylum level composition of OTUs observed in root samples (270 of 345 OTUs were identified). (B) Order-level composition of OTUs observed in root samples (185 of 345 OTUs were identified). (C) Genus level composition of OTUs observed in root samples (112 of 345 OTUs were identified).

Among the 345 fungal OTUs found from the 159 terminal-root samples, 270 (78.3%) were identified to phylum, 185 (53.6%) were identified to order and 112 (32.5%) were identified to genus (Fig. 2). At phylum level, 168 fungal OTUs (62.2%) were ascomycetes, 93 (34.4%) were basidiomycetes, five were glomeromycetes (1.9%), and two were chytridiomycetes (0.7%) (Fig. 2A). At order level, Helotiales, Russulales, and Agaricales dominated the root-associated fungal community, while diverse clades such as Chaetothyriales and Eurotiales were found as well (Fig. 2B). At the genus level, the ectomycorrhizal taxon *Russula* was the most common in the root samples (Fig. 2C). Besides *Russula*, fungi in diverse ectomycorrhizal genera such as *Lactarius*, *Cortinarius*, *Lactarius*, *Tomentella*, *Amanita*, *Boletus*, and *Cenococcum* were observed (Fig. 2C). Meanwhile, we found diverse nonectomycorrhizal fungi such as *Capronia* (= *Cladophialophola* [anamorph]), *Cryptosporiopsis*, *Oidiodendron*, and *Hypocrea*, genera known to include root endophytes and plant pathogens.

Of the 345 OTUs found from roots, 56 were putatively ectomycorrhizal, five were putatively arbuscular mycorrhizal and five were putatively parasitic, while the ecotype of remaining 279 OTUs could not be inferred solely based on their taxonomy (Data S4).

### Properties of dominant root-associated fungi

**Figure 3:**
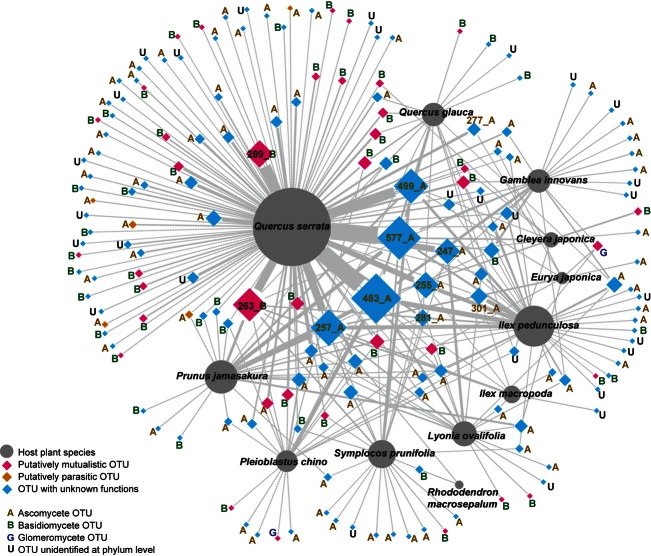
Architecture of the belowground plant–fungal associations. Symbiosis of a plant species (gray circle) with a fungal OTU (diamond) was represented by a line between the symbols. The thickness of links indicates the number of times respective interactions are observed. Putatively mutualistic fungal OTUs (pink), putatively parasitic OTUs (orange), and OTUs with unknown functions (blue) were indicated by the color of symbols. The square measure of nodes roughly represents the relative occurrence of plant species or fungal OTUs in the community. The IDs of the 10 most common OTUs are indicated. Fungi in the phyla Ascomycota (“A”), Basidiomycota (“B”), and Glomeromycota (“G”), as well as fungi unidentified at phylum level are indicated by letters.

The root-associated fungal community was dominated by fungi in the basidiomycete ectomycorrhizal family Russulaceae and by fungi from diverse ascomycete clades (Table 1; Fig. 3). Phylogenetic analysis indicated that the common ascomycetes belonged to three orders that include diverse root-endophytic fungi (Helotiales, Chaetothyriales, and Rhytismatales; Fig. S3; Table 1).

**Table 1:** The 10 most common fungal OTUs in roots

OTU ID	No. obs	Phylum	Class	Order	Family	Genus^1^	Habitat specificity (root vs. soil)	Host specificity (*d'*)	BLAST accession	Description	E-value	Identity (%)
483	63	Ascomycota	Leotiomycetes	Helotiales			Root	0.027	GU166474.1	Helotiales sp.	6E-142	96
577	48	Ascomycota	Eurotiomycetes	Chaetothyriales	Herpotrichiellaceae		Root	0.036	HM803232.1	*Cladophialophora carrionii*	2E-137	93
257	34	Ascomycota	Leotiomycetes	Helotiales			Root	0.098	HM190132.1	Ascomycota sp.	3E-160	100
499	31	Ascomycota	Leotiomycetes	Rhytismatales			Root	0.063	GU138714.1	*Lophodermium jiangnanense*	1E-68	83
263	28	Basidiomycota	Agaricomycetes	Russulales	Russulaceae	*Russula*	Both habitats	0.032	AB597671.1	Fungal sp.	3E-174	97
289	19	Basidiomycota	Agaricomycetes	Russulales	Russulaceae	*Lactarius*^2^	Both habitats	0.173^1^	AB597656.1	Fungal sp.	0	100
255	18	Ascomycota	Leotiomycetes	Helotiales			Too rare^3^	0.095	AF081443.2	Mycorrhizal sp.	3E-159	99
247	17	Ascomycota	Leotiomycetes	Helotiales	Dermateaceae		Too rare^3^	0.124	JN607229.2	Fungal sp.	2E-161	100
281	8	Ascomycota	Eurotiomycetes	Chaetothyriales	Herpotrichiellaceae		Too rare^3^	0.025	GQ996076.1	Fungal sp.	3E-169	98
301	8	Ascomycota	Leotiomycetes	Rhytismatales			Too rare^3^	0.285	FR837916.1	Rhytismataceae sp.	6E-112	91

Frequently observed fungal OTUs are listed in terms of the number of observations in 159 root samples. Number of links to plants, the result of CLAM test (Fig. 3), and *d'* measure of specificity to host plants are shown. Detailed identification of the ascomycete OTUs was performed based on a phylogenetic analysis (Fig. S1). The results of normal BLAST search are also shown with E-values and maximum identity scores.

1 Significantly high specificity to host plants (*P* < 0.05).

2 Taxonomic information was assigned at species level (*Lactarius quietus*).

3 Fungus detected only from root samples; habitat specificity was not assigned by CLAM test due to a small number of observations (Fig. 3).

**Figure 4:**
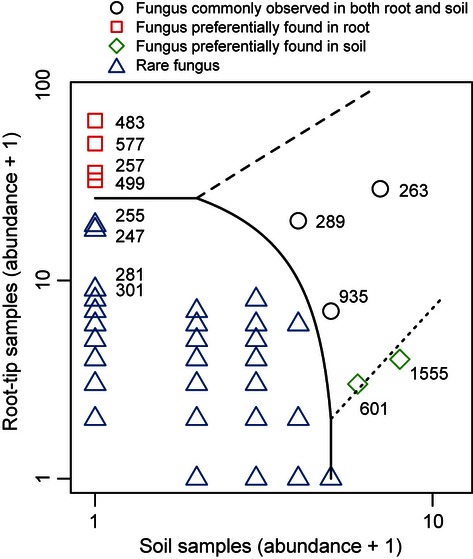
Habitat preference of the observed fungi. Each fungal OTU is plotted along the axes indicating the time of appearance in 38 soil samples and that in 159 root samples. Based on the CLAM test (Chazdon et al. 2011), each OTU was classified as showing statistically significant habitat preference to root samples, to soil samples, or as being commonly observed in both habitats. The 10 most common OTUs in root samples are indicated by their OTU IDs. Note that one was added to original values for log transformation.

The ectomycorrhizal basidiomycete fungus *Lactarius quietus* (OTU 289) was exclusively associated with the dominant plant *Q. serrata*; in contrast, the other common ectomycorrhizal basidiomycete *Russula* sp. (OTU 263) was associated with seven host genera (Figs. 1D and 4; Table 1). All eight common ascomycetes were found in samples from diverse plant species (Fig. 3). Notably, the most common OTU in Helotiales (OTU 483) was detected from 10 of the 12 sampled plant species (Fig. 3). Both common ectomycorrhizal basidiomycetes were found in both root and soil samples at relatively high frequency, and were thus characterized as habitat generalists (Fig. 4; Table 1). In contrast, the eight common ascomycetes were only found in root samples, and four showed statistically significant habitat preference for root over soil (Fig. 4; Table 1; see also Fig. S4 for difference in overall community structure between roots and soil).

### Co-occurrence of fungal OTUs within roots

**Figure 5:**
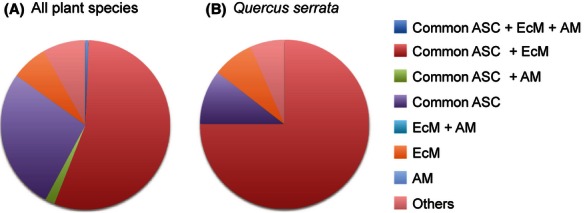
Co-occurrence of mycorrhizal fungi and putatively endophytic ascomycetes within roots. (A) Proportion of root samples within which co-occurrence of different ecotypes of fungi were observed. All the 159 root samples of 12 plant species were examined. (B) Proportion of *Quercus serrata* root samples within which co-occurrence of different types of fungi were observed. All the 76 root samples of *Q. serrata* were examined. Common ASC, the eight most common ascomycetes (putative root endophytes; see text); EcM, ectomytorrhizal fungi; AM, arbuscular mycorrhizal fungi; Others, roots without the common ascomycetes, ectomycorrhizal fungi and arbuscular mycorrhizal fungi.

Of the 159 root samples examined, 84.9% (135/159) were colonized by at least one of the eight common ascomycetes (Fig. 5). Importantly, most of those roots also colonized by an arbuscular or an ectomycorrhizal fungus. Of the 159 root samples, 55.3% (88/159) were colonized by both common ascomycete and ectomycorrhizal fungi, 1.9% (3/159) were colonized by both common ascomycete and arbuscular mycorrhizal fungi, and 0.6% (1/159) were colonized by all the three ecotypes (Fig. 5). Moreover, of the 76 root samples of the dominant plant *Q. serrata*, 75.0% (57/76) were colonized by both the common ascomycetes and ectomycorrhizal fungi; only 7.9% (6/76) were colonized by ectomycorrhizal fungi but none of the eight common ascomycetes.

## Discussion

We found broad patterns of co-occurrence between root-endophytic fungi and mycorrhizal fungi in an oak-dominated temperate forest. Ectomycorrhizal fungi were found both in root samples and in the soil surrounding the roots, reflecting the expected nutrient foraging strategy. In contrast, endophytic ascomycetes were primarily restricted to root samples. The host ranges of endophytic ascomycetes were generally broader than those of ectomycorrhizal basidiomycetes. The structure of the root fungal communities points to the importance of studies to understand how co-occurrence of terminal roots by endophytic and mycorrhizal fungi could influence host plant performance.

The second-growth forest included many fungal clades expected in *Quercus*-dominated North-temperate forests (Jumpponen et al. 2010; Sato et al. 2012a,b; Tedersoo et al. 2012). Basidiomycete fungi in Russulaceae were the most common, while *Cortinarius*, *Tomentella*, *Amanita*, *Boletus*, and the ascomycete *Cenococcum* were also found in root samples (Fig. 2; Table 1). These fungi were found in roots as well as in the surrounding soil, as would be expected from ectomycorrhizal fungi that produce extraradical mycelia to forage for nutrients (Finlay and Read 1986).

Somewhat surprisingly, root-endophytic ascomycetes were more common than ectomycorrhizal basidiomycetes (Fig. 3; Table 1). The fungal community of roots was dominated by ascomycetes in diverse taxonomic clades such as Helotiales, Chaetothyriales, and Rhytismatales (Figs. 3 and S3; Table 1). These common ascomycetes had broad host ranges, as reported in previous studies of root endophytes (Newsham 2011; Knapp et al. 2012; Mandyam et al. 2012). They were also largely restricted to plant tissue, as are many foliar endophytes (Rodriguez et al. 2009). These common ascomycetes constitute a major ecotype that codominates the root-associated fungal community together with ectomycorrhizal basidiomycetes (Fig. 5).

Although the ecological functions of ascomycete root endophytes remain poorly known, experimental inoculations suggested that some could help plant hosts to acquire inorganic form of nitrogen (Upson et al. 2009; Newsham 2011). However, because they rarely appear to produce extraradical mycelia, their roles in nutrient uptake might be different from those of ectomycorrhizal fungi (Read and Perez-Moreno 2003; Smith and Read 2008). They may contribute to the nutrient acquisition of plant hosts by secreting enzymes that degrade organic nitrogen and/or phosphorus to inorganic ones in rhizosphere (Upson et al. 2009; Newsham 2011), but are unlikely to transport nutrients from distant places that are inaccessible by the plant roots (cf. Finlay and Read 1986).

The common pattern of co-occurrence of ectomycorrhizal fungi and ascomycete root endophytes within terminal roots (Fig. 5) suggests that these two ecotypes of fungi are likely to be involved in some kinds of ecological interactions within roots (Wagg et al. 2008). Although functions of root endophytes deserve further ecological and physiological investigations (Newsham 2011; Porras-Alfaro and Bayman 2011), the common pattern of co-occurrence suggests the possibility that interactions between the two ecotypes could be mutualistic or commensal rather than completely neutral. For example, an experimental study showed that the exudates of a dark septate endophyte stimulated the hyphal length and hyphal branching of a mycorrhizal fungus, suggesting that root endophytes could promote the symbiosis between their host plants and mycorrhizal fungi (Scervino et al. 2009). Alternatively, root endophytes themselves can be commensal secondary colonizers of roots (Tedersoo et al. 2009) and they may be attracted by exudates of primary mycorrhizal symbionts. However, similarity in ecological niches (e.g., chemical properties of terminal roots) can also generate a pattern of co-occurrence without any particular interaction between the fungi. Hence, carefully designed experimental studies are needed to further understand the reasons behind the co-occurrence and potential ecological interactions between mycorrhizal and root-endophytic fungi.

In this study, we quantitatively evaluated the community structure of root-associated fungi by sampling plant terminal roots indiscriminately in terms of their morphology and mycorrhizal type. Based on the sampling strategy, the target of this study was not confined to specific ecotypes of root-associated fungi (e.g., ectomycorrhizal fungi) and should roughly represent the belowground community structure of plants occurring in the study site (sensu Hiiesalu et al. 2012). Thus, this sampling method enables the simultaneous investigation of belowground fungal and plant communities, further giving chance to examine the relative frequency of plant–fungal associations in local forests (Fig. 3; Nielsen and Bascompte 2007; Montesinos-Navarro et al. 2012). Consequently, the “community-wide” sampling method would be suited for ecological studies to quantitatively investigate the entire community structure of root-associated fungi in a study site. In contrast, the standard mycological method that targets specific host plant taxa would be more efficient when intensively examining fungal species associated with the focal plants.

On the basis of the massively parallel pyrosequencing, we found that ectomycorrhizal and root-endophytic fungi constituted a complex community in an oak-dominated temperate forest. Although the “codominance” of the two ecotypes was of particular ecological interest, our study reported data from just one location and one point in time. Therefore, to examine whether the codominance of mycorrhizal and root-endophytic fungi is prevalent in nature, we need to conduct community-wide analyses of root-associated fungi in various forests differing in climate and/or vegetation. Furthermore, ecological and physiological functions of root endophytes, and particularly how they interact with co-occurring mycorrhizal fungi, remain to be intensively investigated in experimental studies.

## Supporting Information

**Data S1.** OTU sequences in FASTA format.

**Data S2.** Summary of reads that passed quality filtering.

**Data S3.** Comparison of molecular identification results for the 10 most common OTUs.

**Data S4.** OTUs observed in root and soil samples (all OTUs).

**Data S5.** Matrix representing the presence/absence of each fungal OTU in each root or soil sample.

**Data S6.** Matrix representing the symbiosis of plant species and fungal OTUs.

**Figure S1.** Map of the study plot. Schematic illustration of the sampling design in the study site. Root samples were collected on crossover points of the 1-m-mesh plot. Soil samples were collected on crossover points of the 2-m-mesh plot as indicated by the brown circles. Numbers indicate sample IDs in Data S2.

**Figure S2**. Example photographs of washed terminal roots. Example photographs of washed terminal roots. Each terminal root was washed in 70% ethanol by shaking it with 1-mm zirconium balls 15 times per second for 2 min using TissueLyser II (Qiagen).

**Figure S3.** Example photographs of washed terminal roots. Molecular phylogeny of most commonly observed ascomycete OTUs. Maximum-likelihood topology based on ITS sequences is shown with bootstrap probabilities above the branches (>50%; 100 replicates). Fungal sequences of “dark septate endophytes” (Grünig et al. 2011; Newsham 2011) are indicated by asterisks. (A) OTUs in the order Helotiales (254 bp; J2ef + G model). (B) OTUs in the order Chaetothyriales (239 bp; TN93ef + G). (C) OTUs in the order Rhytismatales (258 bp; TN93ef + G).

**Figure S4.** Molecular phylogeny of most commonly observed ascomycetes OTUs. Comparison of fungal community composition between root and soil samples. (A) Taxonomic composition of OTUs observed in root samples (98 of 172 OTUs identified at the order level). The analysis was conducted after converting the pyrosequencing data to the presence/absence matrix (see Materials and Methods; Data S5). Asterisks indicate OTUs whose order-level taxonomy is yet to be settled, but genus- or family-level taxonomic information is available. (B) Taxonomic information of OTUs observed in soil samples (41 of 90 OTUs identified at the order level). (C) Taxonomic information of OTUs observed in root samples (66 of 172 OTUs identified at the genus level). (D) Taxonomic information of OTUs observed in soil samples (30 of 90 OTUs identified at the genus level).
