# Supplementary figures and images for: Community composition of root-associated fungi in a Quercus-dominated temperate forest: “codominance” of mycorrhizal and root-endophytic fungi
Source: Ecol Evol. 2013 Apr 5;3(5):1281–93. doi: 10.1002/ece3.546 (PMC3678483; doi:10.1002/ece3.546)

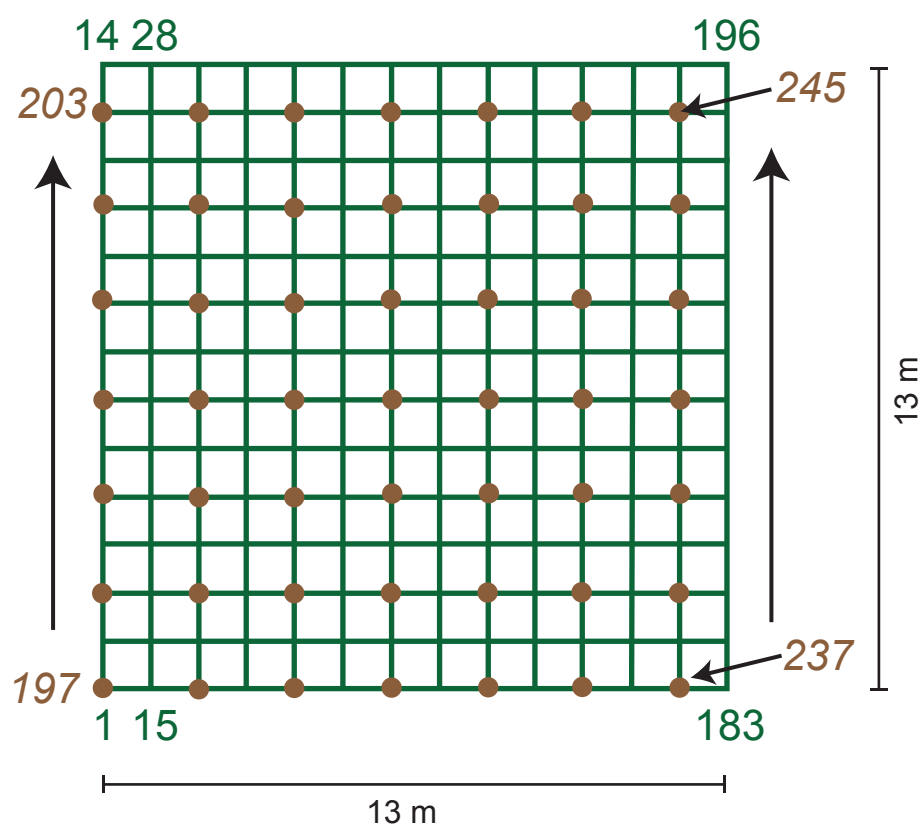

Fig. S1

Supplement: Supplementary file 7 [file ece30003-1281-SD7.pdf]

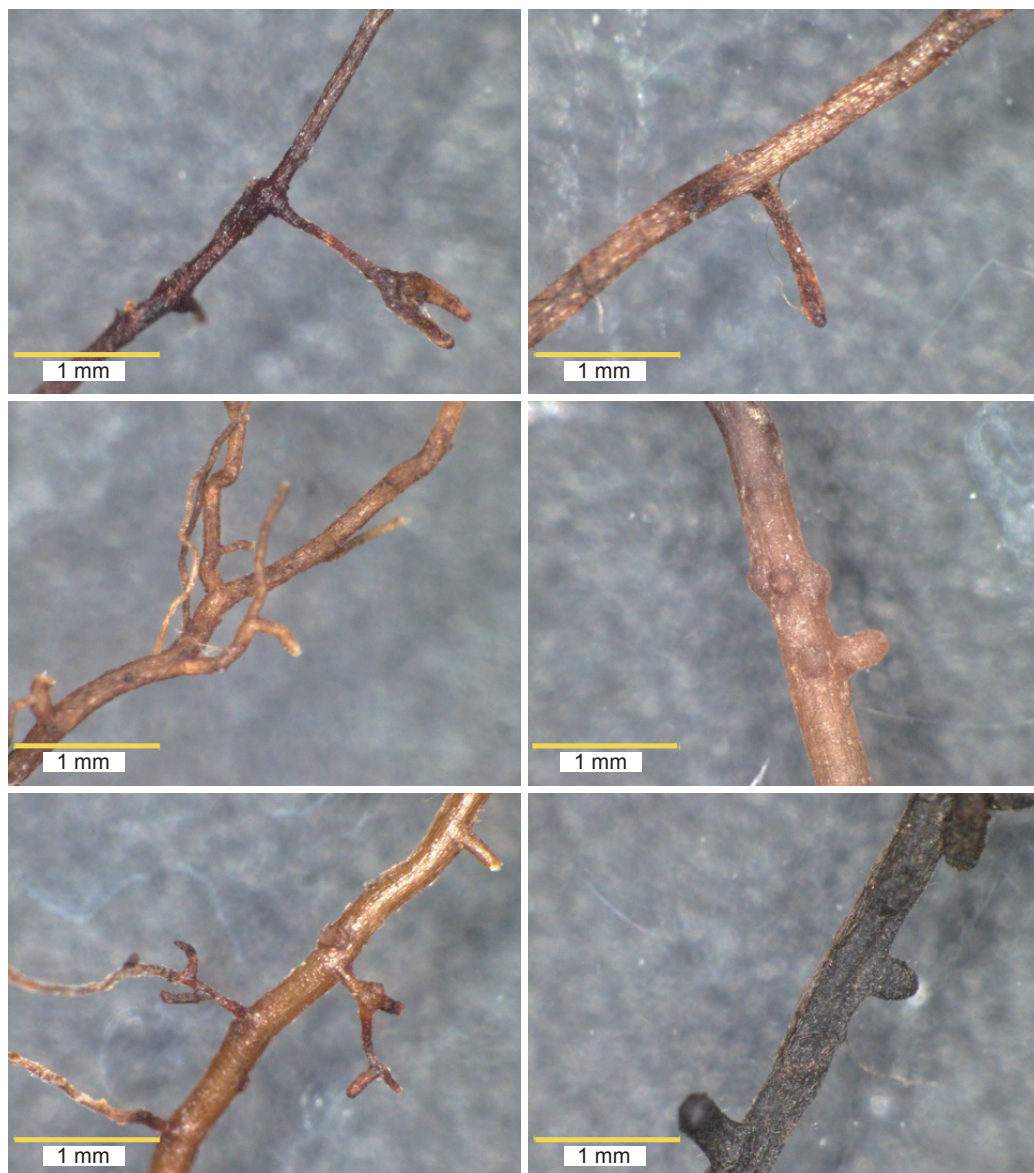

Fig. S2

Supplement: Supplementary file 8 [file ece30003-1281-SD8.pdf]

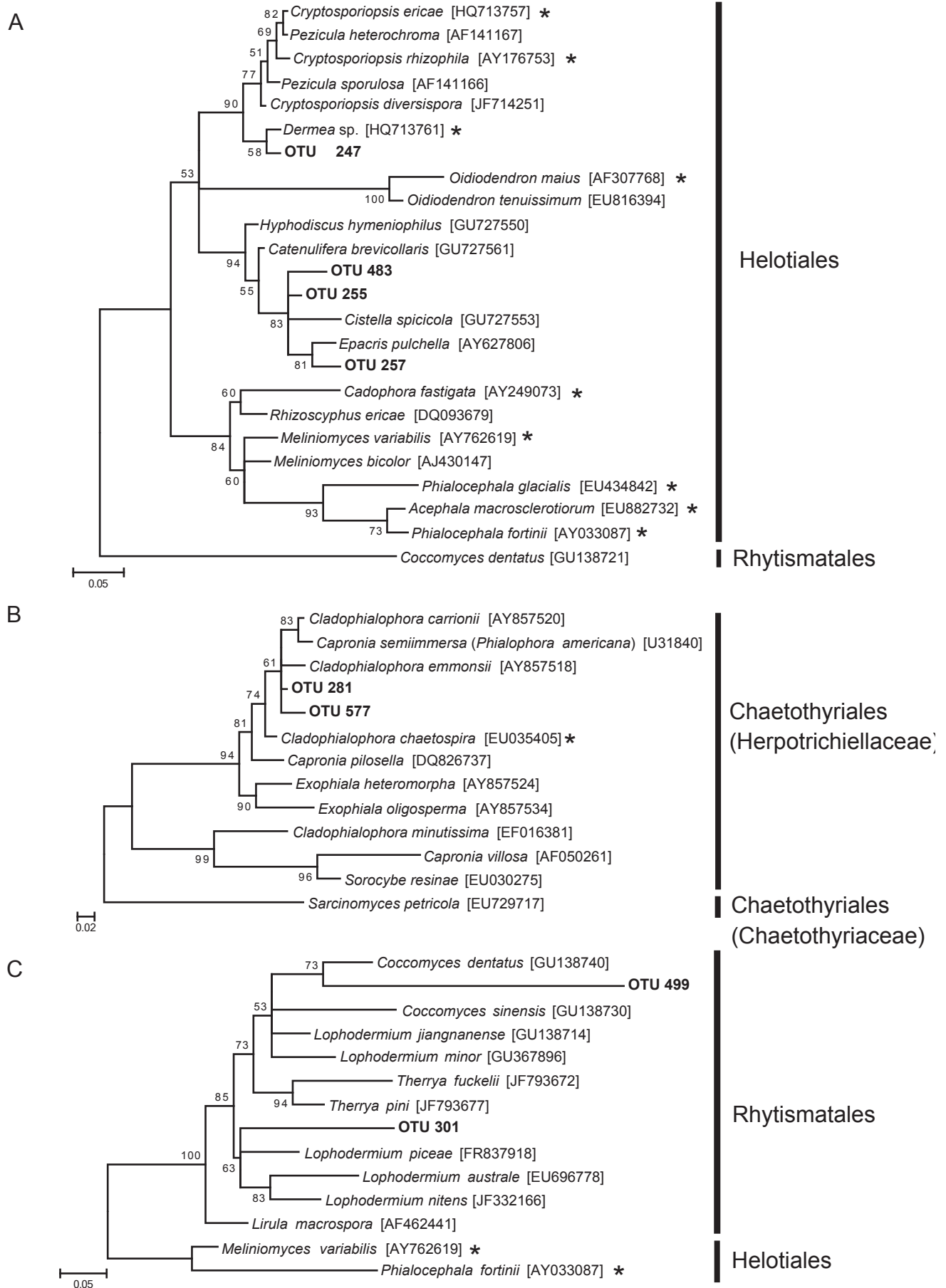

Fig. S3

Supplement: Supplementary file 9 [file ece30003-1281-SD9.pdf]

A. Root (order)

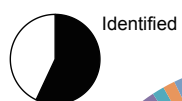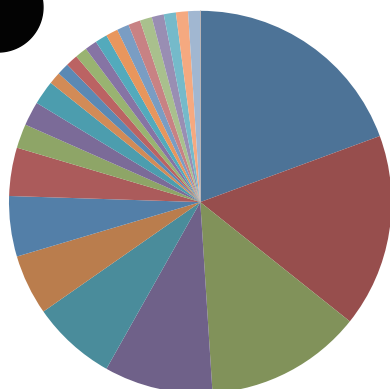

B. Soil (order)

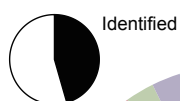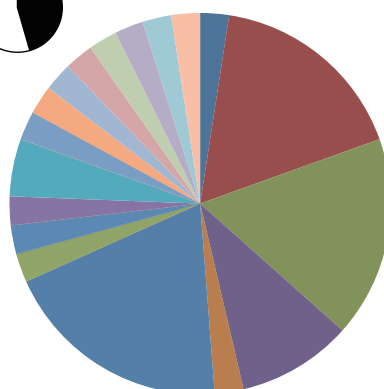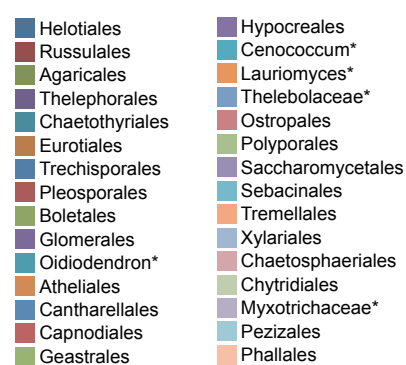

C. Root (genus)

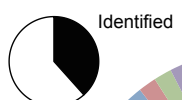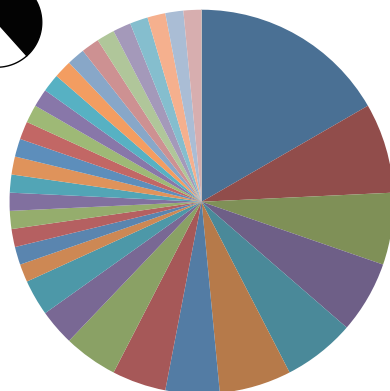

D. Soil (genus)

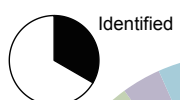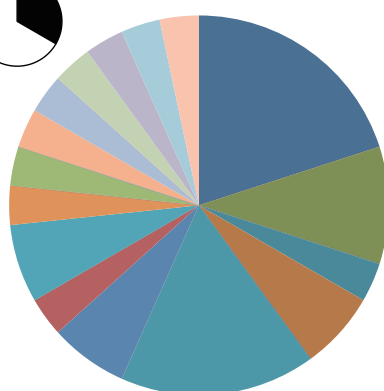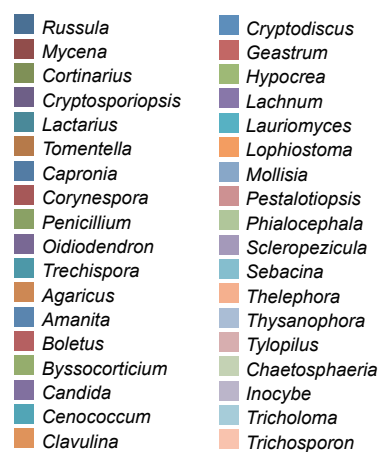

Fig. S4

Supplement: Supplementary file 10 [file ece30003-1281-SD10.pdf]
